# Modeling the Potential of Treg-Based Therapies for Transplant Rejection: Effect of Dose, Timing, and Accumulation Site

**DOI:** 10.3389/ti.2022.10297

**Published:** 2022-04-11

**Authors:** Maya M. Lapp, Guang Lin, Alexander Komin, Leah Andrews, Mei Knudson, Lauren Mossman, Giorgio Raimondi, Julia C. Arciero

**Affiliations:** ^1^ Department of Mathematics, The College of Wooster, Wooster, OH, United States; ^2^ Department of Mathematics, Purdue University, West Lafayette, IN, United States; ^3^ Department of Plastic and Reconstructive Surgery, Johns Hopkins School of Medicine, Baltimore, MD, United States; ^4^ Department of Mathematics, St. Olaf College, Northfield, MN, United States; ^5^ Department of Mathematics, Carleton College, Northfield, MN, United States; ^6^ Department of Mathematical Sciences, Indiana University-Purdue University of Indianapolis, Indianapolis, IN, United States

**Keywords:** regulatory T cells, rejection, heart transplant, mathematical model, adoptive transfer, immune response

## Abstract

**Introduction:** The adoptive transfer of regulatory T cells (Tregs) has emerged as a method to promote graft tolerance. Clinical trials have demonstrated the safety of adoptive transfer and are now assessing their therapeutic efficacy. Strategies that generate large numbers of antigen specific Tregs are even more efficacious. However, the combinations of factors that influence the outcome of adoptive transfer are too numerous to be tested experimentally. Here, mathematical modeling is used to predict the most impactful treatment scenarios.

**Methods:** We adapted our mathematical model of murine heart transplant rejection to simulate Treg adoptive transfer and to correlate therapeutic efficacy with Treg dose and timing, frequency of administration, and distribution of injected cells.

**Results:** The model predicts that Tregs directly accumulating to the graft are more protective than Tregs localizing to draining lymph nodes. Inhibiting antigen-presenting cell maturation and effector functions at the graft site was more effective at modulating rejection than inhibition of T cell activation in lymphoid tissues. These complex dynamics define non-intuitive relationships between graft survival and timing and frequency of adoptive transfer.

**Conclusion:** This work provides the framework for better understanding the impact of Treg adoptive transfer and will guide experimental design to improve interventions.

## Graphical Abstract

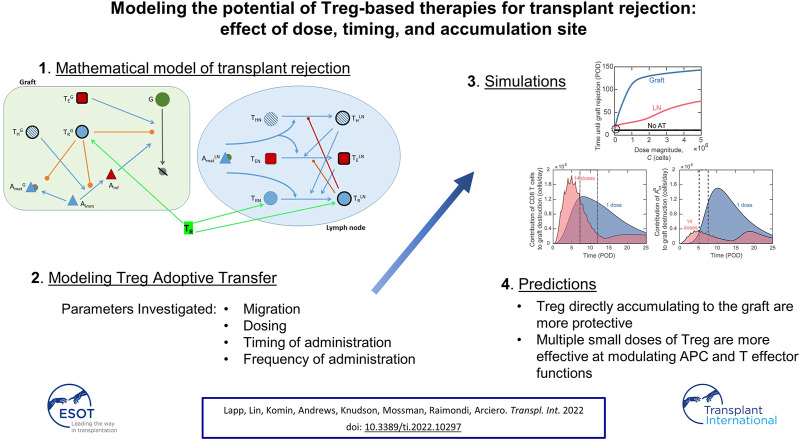

## Introduction

Following transplantation, lifelong immunosuppression is required to prevent allograft rejection (1). Unfortunately, because of drug-associated complications (1–3) and the unfeasibility of complete immunosuppression, 5 years survival rates for patients undergoing solid organ transplantation range anywhere from 40–70%, depending on the organ transplant type (4–6). Thus, there is an urgent need to develop alternative treatment strategies to promote graft tolerance and improve the quality of life for transplant recipients.

One such promising strategy is the adoptive transfer of regulatory T cells (Tregs). Tregs are lymphocytes that suppress the activity of other immune cells and are critical for maintaining peripheral tolerance and preventing autoimmune pathologies (7). The idea is that adoptive transfer of large numbers of Tregs can suppress transplant rejection and promote establishment of tolerance to the transplanted organ. Studies in pre-clinical animal models have shown feasibility and efficacy of polyclonal Treg infusion (8–13). Results from the completed clinical trials provide evidence that delivery of expanded polyclonal Treg is safe (14–16) and possibly effective (17). Excitingly, preclinical studies suggest that the therapeutic effect can be improved by using alloantigen-specific Tregs (18–20). To achieve this, Tregs can be modified to express Chimeric Antigen Receptors (CAR) or transgenic T cell receptors, which endow large-scale production of Tregs with the desired antigen specificity (10, 21, 22). In animal transplant models, CAR-Tregs have been shown to reach the grafts (19, 23, 24) and to control skin graft rejection to a greater extent than polyclonal Tregs (19, 24). CAR-Treg application and safety is currently being investigated in the first clinical trial in Europe (STEADFAST study, Sangamo Therapeutics, Inc.).

Despite these promising outcomes, much remains unknown about the consequences and efficacy of Treg adoptive transfer and what conditions maximize their therapeutic effect. It is difficult to compare experimental outcomes between studies due to differences in model organisms, transplanted organs, dose magnitude, dose timing, and Treg population quality (affected by *in vitro* expansion). Moreover, disparities in the results of some Phase I clinical trials highlight this lack of understanding of their optimal use (13, 25, 26). Further clinical trials will continue to improve our comprehension of adoptive transfer efficacy. However, it takes years to evaluate the long-term effects of this treatment, and development of methods to facilitate a more rapid understanding of the impact and optimization of treatment regimen would be invaluable.

Mathematical and computational models have been widely used in conjunction with experimental methods in cancer and virology to understand immune system dynamics and aid in the design of effective immunotherapies (27–29), but their application in transplantation is lacking. Some theoretical models for solid organ transplant rejection have been proposed (30–35), several of which focused on the impact of immunosuppression only (31, 33–35) and used simplified representations of immune components. We established one of the first theoretical models to describe the immune system and transplant dynamics that give rise to transplant rejection, and ours is currently the only transplantation model using differential equations to track Tregs independently from other T cell populations (30). Both our work and that of De Gaetano et al. incorporated experimental methods in developing transplant rejection models (30, 31). However, no mathematical model to date provides a robust mechanism for analysis of Treg adoptive transfer on the immune response to transplantation.

In the present study, we adapted our model (30) to include new equations and terms to simulate the impact of alloantigen-specific Treg adoptive transfer on graft survival (Supplementary Figure S1). We updated the model equations for antigen-presenting cells (APCs) to match their behavior observed *in vivo* (36). We also performed parameter estimation on the updated model using a non-dominated sorting-based multi-objective evolutionary algorithm (37), called NSGA-II (Non-dominated Sorting Genetic Algorithm II). Although adoptive transfer is not sufficient to prevent graft rejection independently of immunosuppression (10), in this study we focus exclusively on adoptive transfer treatments to elucidate their direct effect on transplant rejection without the complicating and possibly confounding effects of concomitant immunosuppression. Thus, using the updated model, this study aimed at: 1) identifying optimal conditions for Treg delivery, specifically the activation status and tissue distribution, magnitude of dose, timing of delivery, and frequency of dosing, 2) analyzing immune dynamics to explain the effects of adoptive transfer on graft survival, and 3) suggesting future avenues for experimental studies into adoptive transfer treatment.

Our model simulations and analysis identified that timely inhibition of dendritic cell (DC) maturation in the graft and prolonged modulation of cytotoxic CD8 T cells and inflammatory macrophage activity in the graft by Treg were significantly more impactful than inhibiting the activation of alloreactive T cells in draining lymphoid tissues. Use of the model allowed us to identify a non-intuitive correlation between Treg dosing and administration frequency that delineates more effective interventions. Overall, our model enables the rapid simulation of a vast number of conditions that would be prohibitive to cover experimentally. It also provides the framework for future modeling efforts that will assess combinatorial treatments involving both Treg adoptive transfer and immunoregulatory agents.

## Materials and Methods

This study does not involve any new animal or human studies, and thus it was exempt from IRB approval.

### Model Description

We expanded our previously developed model describing the dynamics of murine heart transplant rejection (30) to examine the impact of adoptive transfer of Tregs on graft survival. The original model consists of 13 nonlinear first order ordinary differential equations (ODEs) tracking the following populations in a representative lymph node compartment (LN) and graft compartment (G): CD4 T cells ( $T_{H}^{LN}$ and $T_{H}^{G})$ , CD8 T cells ( $T_{E}^{LN}$ and $T_{E}^{G})$ , regulatory T cells ( $T_{R}^{LN}$ and $T_{R}^{G})$ , immature DCs $\left(A_{imm}\right)$ , mature DCs ( $A_{mat}^{LN}$ and $A_{mat}^{G})$ , inflammatory macrophages ( $A_{inf}$ ), pro- and anti-inflammatory cytokines ( $C_{p}$ and $C_{a})$ , and graft cells ( G ). An additional ODE is introduced to track naïve Tregs ( $T_{RN}^{LN}$ ). All model equations and parameters appear in the Supplemental Digital Content, and all model variables and their initial values are provided in Supplementary Table S1. A complete list of model assumptions is provided in (30). While this model is parametrized for mouse heart transplantation, the predicted interactions between the immune response and transplanted organ would be similar for other transplants.

In this study, the model equations tracking the immature and mature DCs are adapted to include more realistic representations of experimental observations (36). Specifically, the decay rate of immature DCs is assumed to depend on the remaining graft mass (Supplementary Eq. S10, second term). In addition, DC maturation is assumed to occur in the presence of pro-inflammatory cytokines or CD4 T cells (Supplementary Eq. S10, third term; Supplementary Eq. S11, first term). For verification of the modified model and updated predictions for host immune dynamics without adoptive transfer treatment, see the Supplemental Digital Content (Supplementary Figures S2, S3).

**FIGURE 1:**
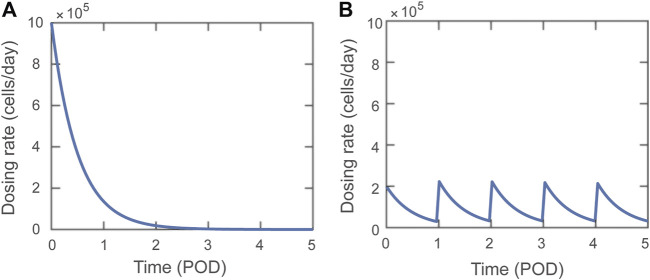
Visual representations of Dosing function, D(t) , delivering a total of $C\;=\;5\;\times 10^{5}$ Tregs. **(A)** A single dose with dosing rate $D_{0}=10^{6}$ cells/day. **(B)** Five doses delivered on POD0-4 with dosing rate $D_{0}=\frac{10^{6}}{5}$ cells/day.

A dosing function, D(t) , for the adoptive transfer of Tregs is defined in Eq. 1 (also in Supplementary Eq. S12). Parameters $f_{G}$ , $f_{LN}$ , and $f_{N}$ correspond to the fraction of the Treg dose that enters the graft as activated Tregs (Eq. 2), the lymph node as activated Tregs (Eq. 3), and the lymph node as naïve Tregs (Eq. 4), respectively. For simulations involving adoptive transfer, $f_{G}+f_{LN}+f_{N}=1$ . These three parameters are set to zero when adoptive transfer is not simulated. The dosing function is composed of one or multiple exponentially decaying functions of time, where *D*_*0*_ indicates the dosing rate, *n* indicates the total number of doses delivered, $t_{i}$ indicates the post-operative day (POD) of delivery of the *i*th Treg injection, and β indicates the decay rate. A decay rate of β=2 day^−1^ is used to simulate the relatively fast absorption of Tregs during adoptive transfer. We assume around 50% of injected cells are lost or distribute in non-draining lymphoid tissues (which are excluded from the model), and we define the dose magnitude C to be the total number of injected cells that localize to the modeled lymph node and graft compartments. Thus, for the majority of this study, we simulate adoptive transfer with $C\;=\;5\;\times 10^{5}$ Tregs. The dose magnitude is the area under the curve of our dosing function, which is given by $C=\;\overset{\infty }{\underset{0}{\int }}\left(D_{0}\overset{n}{\underset{i=1}{\sum }}d_{i}\left(t\right)\right)\;dt=\;\frac{nD_{0}}{\beta }$ cells. A single dose with $D_{0}=\;10^{6}$ cells/day or n doses with $D_{0}\;=\;\frac{10^{6}}{n}\;$ cells/day both administer a total of $C\;=\;5\;\times 10^{5}$ Tregs. Figure 1 shows the dosing rate over time for a single dose administered on POD0 with $D_{0}=10^{6}\;$ cells/day (Figure 1A) and 5 doses administered on POD0, 1, 2, 3, and 4 with $D_{0}=\frac{10^{6}}{5}$ cells/day (Figure 1B). The dose magnitude C is varied during model analysis.

$$D\left(t\right)=\;D_{0}\Sigma_{i=1}^{n}d\left(t\right)\;,\;where\;\;d_{i}\left(t\right)=\{\begin{array}{c} 0\;,\;\;t<t_{i}\\ \;e^{-\beta \left(t-t_{i}\right)\;},\;\;t\geq t_{i} \end{array}$$ (1)

$$\frac{dT_{R}^{G}}{dt}=ke_{R}T_{R}^{LN}-\mu_{R}T_{R}^{G}+\frac{r_{RG}T_{R}^{G}\left(T_{E}^{G}+T_{H}^{G}\right)}{\alpha_{6}+T_{R}^{G}}+f_{G}D\left(t\right)$$ (2)

$$\frac{dT_{R}^{LN}}{dt}=\frac{a_{R}T_{RN}^{LN}A_{mat}^{LN}}{\gamma_{2}+A_{mat}^{LN}}-\mu_{R}T_{R}^{LN}+\frac{r_{R}T_{R}^{LN}\left(T_{E}^{LN}+T_{H}^{LN}\right)}{\alpha_{2}+T_{R}^{LN}}-e_{R}T_{R}^{LN}+f_{LN}D\left(t\right)$$ (3)

$$\frac{dT_{RN}^{LN}}{dt}=\mu_{RN}\;\left(T_{0}\;-\;T_{RN}\right)+f_{N}D\left(t\right)$$ (4)

### Model Simulations

The activation state, accumulation site, magnitude, timing, and frequency of adoptive transfer are varied in the model to simulate the impact of each of these factors on the immune response to the graft. Parameters $f_{G}$ , $f_{LN}$ , and $f_{N}$ are varied to determine the impact of activation state (i.e., naïve or activated) and accumulation site of Tregs. The effects of dose magnitude are assessed by varying C . The timing and frequency of Treg doses are evaluated by varying the start day of the dose ( $t_{0}$ ) and the number of doses, *n*. If multiple doses are administered, it is assumed that the doses are given at 1-day intervals. Following our previous work, graft rejection is defined as a 75% reduction in the original number of graft cells (30). The time at which the model predicts graft rejection is used in this study to identify optimal dosing strategies for the adoptive transfer of Tregs.

### Parameter Estimation

To calibrate the model parameters in the updated model (see parameters shaded in Supplementary Table S2), we employed a non-dominated sorting-based multi-objective evolutionary algorithm (37), called NSGA-II (Non-dominated Sorting Genetic Algorithm II), which can greatly improve the efficiency in constrained multi-objective optimization tasks. NSGA-II is a popular non-domination based genetic algorithm for multi-objective optimization and parameter estimation. NSGA-II improves elitism and there is no need to choose sharing parameters a priori.

## Results

### Impact of Treg Accumulation Site

To analyze the effect of Treg adoptive transfer using our model, we first evaluated the impact of the activation status of the injected cells on graft survival. It is well recognized that pre-activated Treg (even after resting) are an order of magnitude more suppressive than naïve Tregs (38, 39). For this analysis, we simulated the equivalent of delivering $C\;=\;5\;\times 10^{5}$ Tregs on POD0. As expected, accumulation of naïve Tregs into the lymph node was not as effective as the adoptive transfer of pre-activated Tregs (Supplemental Digital Content, Supplementary Figure S4). This confirmed the benefit of using *ex-vivo* expanded Tregs, which is a necessary step in most clinical preparations of antigen-specific cells that renders homogeneous populations of pre-activated Treg. For the remainder of the study, we simulated the use of pre-activated Tregs.

**FIGURE 2:**
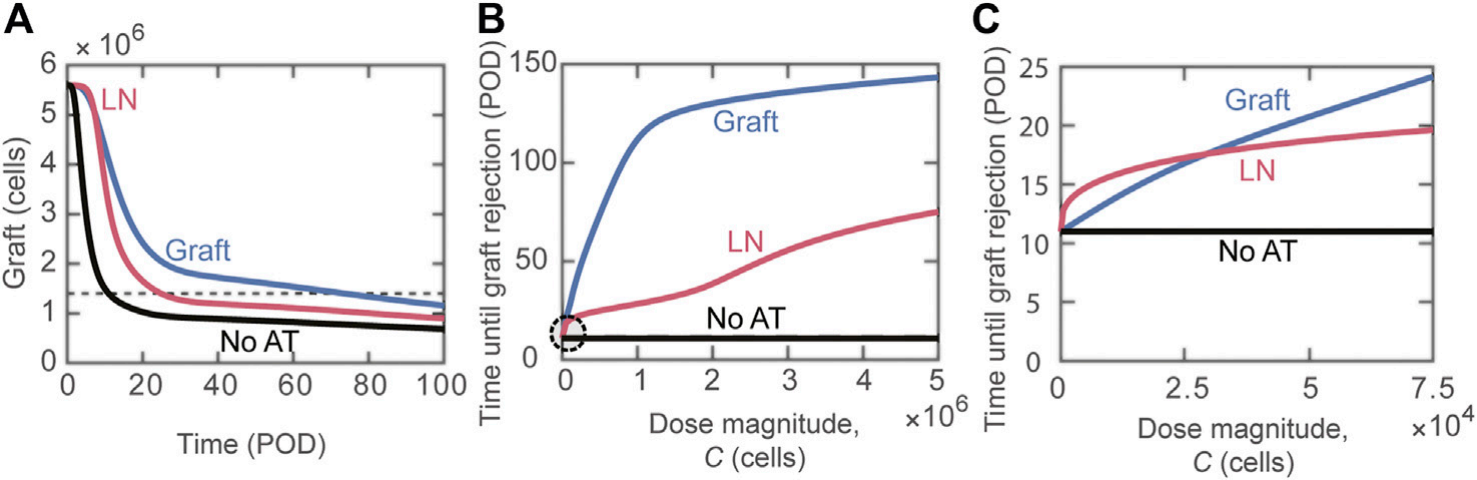
Impact of distribution of adoptively transferred Tregs. **(A)** Number of graft cells shown over time for three different cases: activated Tregs administered to the graft (blue), activated Tregs administered to the lymph node (red), and no Tregs administered (black). Tregs are administered on POD0 with dose magnitude $C\;=\;5\;\times 10^{5}$ cells. The horizontal dashed line indicates a 75% reduction in initial graft size. The intersections of the curves with this dashed line give the model predicted values of graft rejection time. **(B)** Model predicted rejection time as Treg dose magnitude ( C ) is varied. The different curves depict predictions for the delivery of activated Tregs to the graft (blue) and activated Tregs to the lymph node (red). The black line marks POD11, which is when rejection occurs without adoptive transfer. **(C)** Circled region in panel B is magnified to examine the effect of small dose magnitudes on graft rejection.

Next, we focused on the question: does the tissue distribution of Tregs between the lymphoid compartment and the graft post-adoptive transfer impact transplant survival? Figure 2A depicts the change in the graft mass over time for the scenarios where the transferred Tregs exclusively accumulate in the lymphoid versus graft compartments, as well as for the case of no adoptive transfer (black curve). There is a substantial benefit with the accumulation of Tregs in the graft in comparison to lymphoid tissues, with estimated rejection on POD74 vs. POD25, respectively. The model can simulate a wide range of Tregs dose magnitudes; for almost all ranges, accumulation of Tregs directly to the graft (Figure 2B, blue curve) is more effective at extending survival than localization to the lymph node compartment (Figure 2B, red curve). For very small doses ( $C<2.9\times 10^{4}$ cells), delivering activated Tregs to the lymph node is predicted to be more effective than delivering to the graft (Figure 2C); though, it improves graft survival by no more than 2.4 days.

### Impact of Single Dose Timing

**FIGURE 3:**
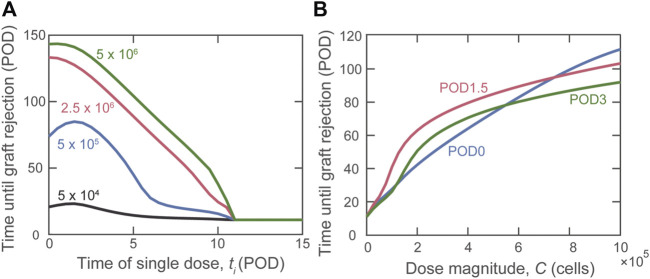
Impact of the timing of Treg administration on graft rejection. **(A)** Predicted graft rejection time as the day of dose administration (*t*_i_) is varied given four different dose magnitudes: $C\;=\;5\;\times 10^{4}$ (black), $C\;=\;5\;\times 10^{5}$ (blue), $C\;=\;2.5\;\times 10^{6}$ (red), and $C\;=\;5\;\times 10^{6}$ (green) cells. **(B)** Predicted graft rejection time as dose magnitude ( C ) is varied for Tregs administered on POD0 (blue), POD1.5 (red), and POD3 (green).

Having shown the benefit of graft localization, we studied the impact of the timing of a single injection of varying amounts of Tregs. Figure 3 shows a non-monotonic relationship between rejection time and day of Treg administration, observed for some Treg dose magnitudes (Figure 3A). Specifically, when using $C\;=\;5\;\times 10^{5}$ cells (blue curve), we observed that delaying Treg administration to POD1.5 yields optimal graft survival time. The non-monotonic behavior between dose timing and graft survival is highlighted for physiological dose magnitudes ( $C<10^{6}$ cells) in Figure 3B. Once the dose magnitude is increased above $C\;=\;7.5\;\times 10^{5}$ cells, a monotonic relationship is re-established in which graft survival time decreases with the delay of Treg administration.

**FIGURE 4:**
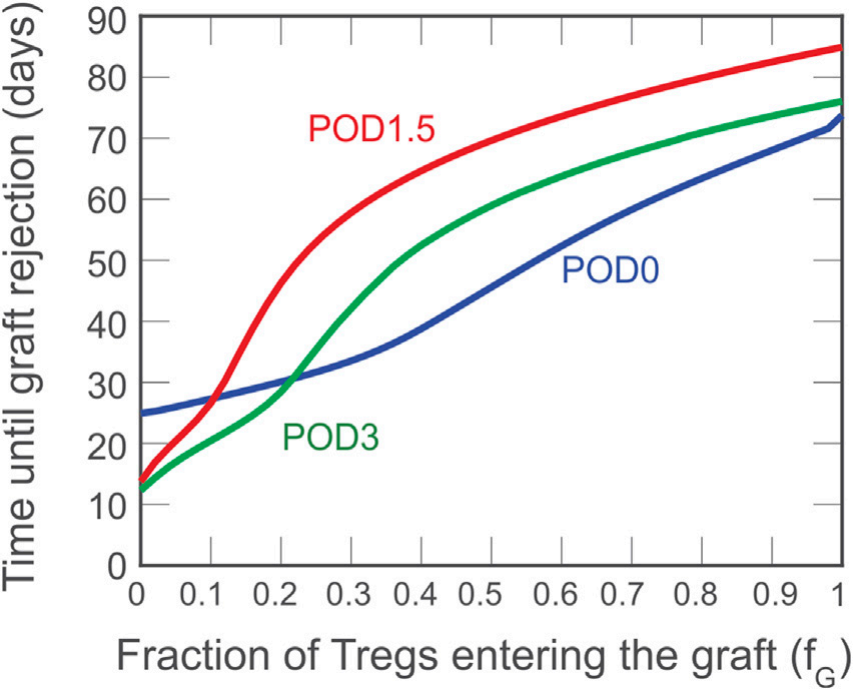
Impact on rejection time of the fraction of activated Tregs that enters the graft. A dose of $C\;=\;5\;\times 10^{5}$ cells is administered on POD0 (blue), POD1.5 (red), and POD3 (green) and the impact on graft survival is projected in relation to the distribution of the injected Treg between graft and lymphoid compartments.

Although a theoretical model can assume that all Tregs administered to an individual accumulate exclusively in the graft or lymphoid tissues, physiological *in vivo* constraints and Treg properties dictate a variable partitioning between the two compartments. Thus, in Figure 4, we show the simulated impact of varying the fraction of Tregs that enter the graft (*f*_*g*_) between 0 and 1; the remaining fraction (1–*f*_*g*_) is assumed to enter the draining lymphoid tissue compartment. As indicated by the red curve, the model predicts that if more than 10% of the injected Tregs locate to the graft, delaying injection until POD1.5 is the most beneficial strategy to prolong the time to graft rejection.

### Impact of Multiple Doses

**FIGURE 5:**
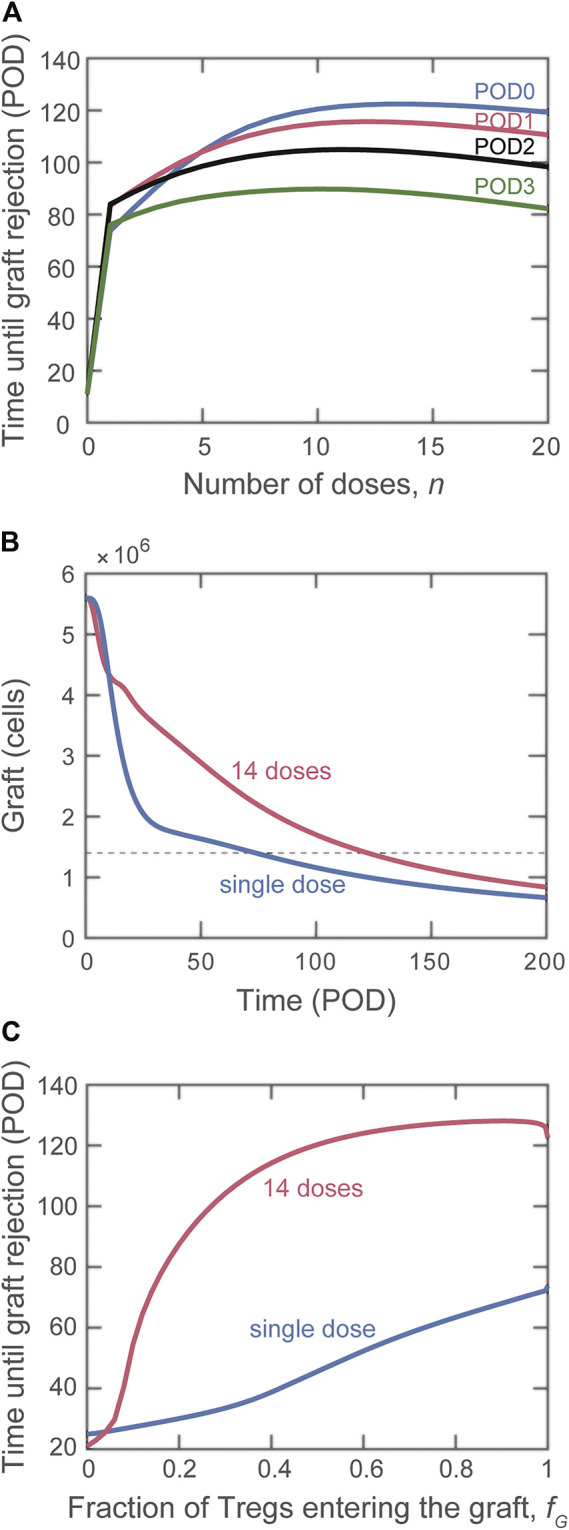
Impact on graft rejection of splitting the same total number of Treg into multiple doses. **(A)** Plot of the simulated rejection time as a function of the indicated number of doses administered starting on POD0 (blue), POD1 (red), POD2 (black), and POD3 (green). The total dose magnitude for each simulation is $C\;=\;5\;\times 10^{5}$ cells. **(B)** Number of graft cells as a function of time for a single dose (blue curve) and for the optimal case of multiple doses (14 daily doses of Tregs beginning on POD0, red curve). **(C)** Comparison of rejection time for single (blue curve) and multiple (red curve) dose administration if the fraction of Tregs that reach the graft is varied.

The model allows us to compare the protective effect of a single injection of $\;C\;=\;5\;\times 10^{5}$ Tregs versus splitting that total number of cells among multiple daily injections (an approach that can also represent sustaining the presence of a smaller amount of Treg over time). As depicted in Figure 5, distributing a fixed dose prolongs graft survival. The relationship between the number of consecutive doses and graft survival was not monotonic, with graft survival peaking around 10–15 equivalent doses, depending on the first day of injection (Figure 5A). Significantly, when enough doses are administered, delaying the start of adoptive transfer treatment is no longer beneficial. The model predicts that administering $C\;=\;5\;\times 10^{5}$ cells divided evenly among 14 daily doses beginning on POD0 is the most effective treatment, extending graft survival until POD115. As shown in Figure 5B (graft mass over time), it is noteworthy that in addition to extending transplant survival, multiple doses of Treg provide better protection of the graft (i.e., slower rate of mass decline). Figure 5C shows that the impact of multiple doses compared with a single dose is apparent even if only a small fraction of Tregs (>5%) enters the graft.

### Impact of Treg Adoptive Transfer on Host Immune Dynamics

**FIGURE 6:**
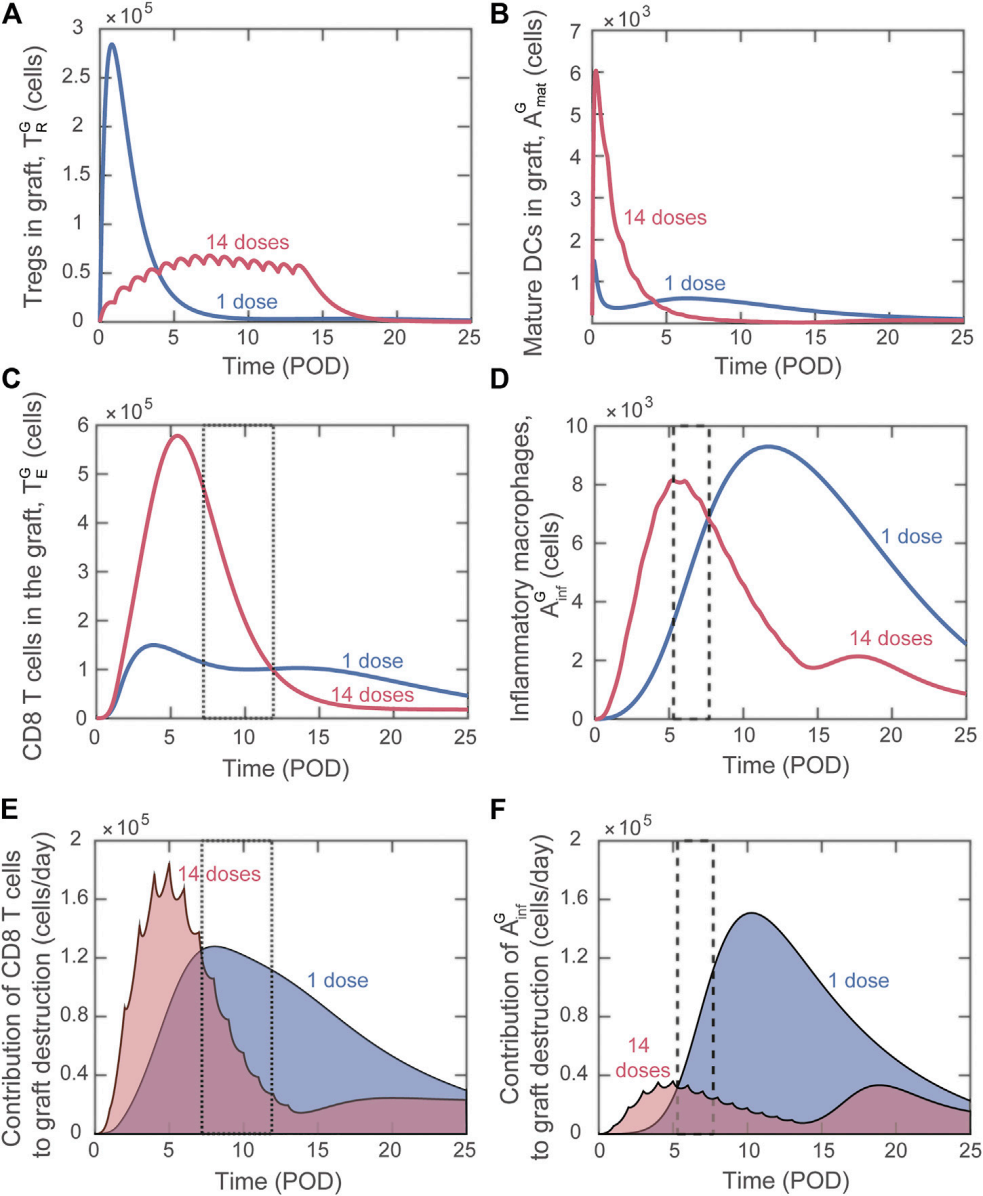
Model predicted alterations of immune dynamics induced by $C=5\times 10^{5\;}$ Tregs delivered as a single dose (blue) or distributed across 14 doses (red). **(A)** Number of Tregs in the graft, $T_{R}^{G}$ . **(B)** Number of mature DCs in the graft, $A_{mat}^{G}$ . **(C)** Number of cytotoxic CD8 T cells in the graft, $T_{E}^{G}$ . **(D)** Number of inflammatory macrophages in the graft, $A_{inf}^{G}$ . **(E)** Rate of graft destruction caused by cytotoxic CD8 T cells. **(F)** Rate of destruction caused by inflammatory macrophages. Dashed boxes [in **(C–F)**] highlight timeframes when indicated destructive cell quantity is higher for the 14 dose treatment but the resulting rate of destruction is lower than for the single dose treatment.

Differently from bioinformatic approaches (e.g., machine learning), mathematical modeling enables the analysis of the dynamics of host immune cells that determine the simulated outcome. This valuable property can be used to gain insight into why certain adoptive transfer treatments are more effective than others. We investigated in detail the difference in effects between using a single injection of graft-infiltrating Tregs versus splitting the total number over multiple injections. Figure 6 depicts the predicted dynamics of host immune cells in the graft with single dose adoptive transfer (blue) and 14 dose adoptive transfer (red). For a similar investigation into the impact of site of accumulation and timing of single injections, please see the Supplemental Digital Content (Supplementary Figures S5, S6).

Delivering a large single dose of Tregs minimizes the maturation of graft infiltrating DCs (Figure 6B) and therefore limits the activation and graft accumulation of cytotoxic CD8 T cells (Figure 6C) and delays the activation of macrophages (Figure 6D). Therefore, delivering a substantial portion of Tregs soon after transplantation is effective at limiting the early inflammatory response. Nevertheless, the regulation of the destructive capacity of those cells is not sustained with a single administration.

When the injected cells are split among 14 doses, elevated Treg levels are maintained during the surge of cytotoxic T cells and inflammatory macrophages (Figures 6A,C,D, red). In contrast, for single dose adoptive transfer, Tregs in the graft decay before the surge of these graft destructive subsets (Figures 6A,C,D, blue). The lack of Treg-mediated control in the last scenario allows each cytotoxic T cell and inflammatory macrophage to cause greater graft damage despite a lower accumulation level. This phenomenon is highlighted in the boxed regions in Figures 6C–F. We note that Figures 6E,F show the value of each term on the right-hand side of the differential equation for graft cells that contributes to graft destruction (Supplementary Eq. S9). Therefore, higher values of the curve in panel E or F indicate a greater contribution to graft destruction. For example, from POD7.2 to POD11.9, although more cytotoxic T cells are predicted to be present with the 14-dose regimen (Figure 6C; the 14-dose curve is above the 1-dose curve in the dotted boxed region), their effector functions are regulated and less graft destruction occurs (Figure 6E; the 1-dose curve is above the 14-dose curve). Similarly, from POD5.3 to POD7.7, although more inflammatory macrophages are predicted with the 14-dose regimen (Figure 6D), less graft destruction is mediated by this subset (Figure 6F).

Overall, analyzing the population dynamics for various Treg dose frequencies indicates that, to optimize adoptive transfer, cell delivery must 1) maintain elevated Treg levels during the surge of cytotoxic T cells and inflammatory macrophages in the graft and 2) deliver enough Tregs early after transplantation to inhibit DC maturation and therefore limit T cell activation. These same principles hold true when examining dose timing and can explain why administering a single dose on POD1.5 is more effective than delivering a single dose on POD0 (see Supplemental Digital Content, Supplementary Figure S6). When examining site of Treg accumulation, minimizing DC maturation is again critical to understand the benefit of delivering Tregs to the graft rather than the lymph node (see Supplemental Digital Content, Supplementary Figure S5).

## Discussion

In this study, we expanded our previous mathematical model of murine heart transplant rejection to incorporate the impact of Treg adoptive transfer. Importantly, and complementary to bioinformatic approaches (40–43), we took advantage of the power of mathematical modeling to enable analysis of the immune dynamics underlying the results of simulations and thereby offer rationales for the therapeutic optimizations proposed. With the advent of feasible and reliable approaches of genetic engineering to generate high numbers of antigen-specific Treg for adoptive transfer (10), our data suggest important optimizations that would maximize the therapeutic efficacy of these cells.

### Impact of Treg Accumulation Site

Our model predicts that Tregs accumulating to the transplant are more therapeutically effective than Tregs distributing to the lymph node (Figure 2). Importantly, analysis of immune dynamics provides the rationale behind such a different outcome: inhibition of DC maturation in the graft is more effective than direct inhibition of T cell activation in the draining lymphoid tissue at minimizing the number of CD8 and CD4 T cells that reach the graft (Supplemental Digital Content, Supplementary Figure S5). Interestingly, experimental reports in rodent models have suggested that an immediate accumulation of Treg in the graft promotes longer survival (44, 45). Overall, our analysis suggests that identifying methods to increase the fraction of Tregs that translocate early on to the graft will maximize the impact of adoptive transfer. It is noteworthy that the conditions of *ex-vivo* Treg expansion impart specific migratory capacity to the cells and these differences impact the therapeutic result obtained (45–49). Moreover, the breadth of genetic engineering that has become possible for clinical products (e.g., CRISPR/Cas9 based modifications) could provide useful tools to impart the ideal behavior in adoptively transferred Tregs.

### Impact of Single Dose Timing

When administering a single dose of Tregs, our simulations suggest that delaying cell delivery is more effective than administering cells on the day of transplantation (Figure 3B). The justification for this unexpected result is similar to that given for splitting Treg into multiple doses. Slightly delaying administration allows Tregs to directly inhibit the destructive functions of cytotoxic T cells and inflammatory macrophages while ensuring a timely reduction of DC activation and, therefore, T cell activation (Supplemental Digital Content, Supplementary Figure S5). Obviously, this scenario is specific to the unique conditions of administering Tregs without any additional manipulation of the recipient (like immunosuppression, see below), but it highlights the impact of examining cellular interactions at the system level.

### Impact of Multiple Doses

The model predicts that maintaining a prolonged influx of Tregs is the most effective method of lengthening graft survival. Delivering multiple, smaller doses of Tregs preserves the graft longer than delivering the same total number of Tregs in a single bolus. This regimen allows for elevated levels of Tregs to remain in the graft during the surge of graft-destructive cells (and control them), while also compromising on an “early enough” limitation of DC maturation. Although the clinical implementation of multiple daily dosing of Treg is unrealistic, this result highlights the important point of sustaining the survival and function of transferred Tregs. This is a contentious issue with the reported negative effect that many immunosuppressive drugs have on the homeostasis and function of Treg (50, 51). There is growing interest in devising approaches to sustain the persistence and function of Tregs. The use of IL-2/anti-IL2 complexes, IL-2 muteins, as well as the genetic engineering of Tregs to respond to “orthogonal” IL-2 are all examples of active investigations to promote Tregs persistence (10, 20, 52). In parallel, the utilization of biomaterials to promote the sustained accumulation of Tregs in proximity to the transplant represent a very promising strategy (53). Our model results provide the rationale to strongly support these ongoing efforts.

### Limitations, Parallelisms, and Future Work

While the processes of sensitivity analysis and parameter estimation performed in this study (based on experimental data) have improved the accuracy of several model parameters, some parameters remain uncertain. For example, the persistence and proliferation of Tregs in the graft are unquantified variables that have a profound impact on the dynamics of graft infiltrating immune cells. Similarly, the relationship between number of Tregs injected and the number of cells that reach the graft is not quantified; the factors that influence such a relationship are poorly defined, and thus, further experimentation is needed (54).

While ours is the only ODE transplantation model to date that tracks Tregs independently of other T cells, several immunological mathematical models have recently been developed to elucidate the role of Tregs on self-tolerance and to identify key Treg interactions with other immune populations (55–58). The assumptions from these models may be used to improve our existing model to better replicate Treg behavior. In particular, given the importance of IL-2 to Treg survival, proliferation, and function, explicitly tracking IL-2 concentrations as in (57) would strengthen our current model and allow further investigation into IL-2 therapies as a method of extending Treg survival.

Although there is very limited quantitative information, other pre-clinical transplant models show important concordance with some of our model simulations. In a mouse model of pancreatic islet transplantation (45), Zhang et al. compared the i.v. infusion of Treg with the co-transplantation at the site of grafting. The significantly longer survival obtained in the latter case agrees with our model-suggested principle of a higher therapeutic impact when Treg can rapidly and directly modulate graft immune populations. Unfortunately, their report did not present different doses or timing of Treg administration. A similar scenario emerges from reports using Treg in mouse skin transplant models. The use of CAR-Treg (59), cell lines of Treg (60), or polyclonal Treg (61) injected on the day of the transplant promotes only a modest increase in transplant survival, a result that correlates with simulation of a fixed number of Treg that probably mostly home to the lymphoid compartment. In all cases, the combination with so called “adjunct therapies” (ranging from thymectomy and T cell depletion to irradiation and bone marrow co-transplantation) is demonstrated as necessary to achieve lasting therapeutic effects. Currently, we can only draw qualitative comparisons to these experimental models since theoretical model parameters would need to be adapted to each different transplant model.

Overall, our modified model is beneficial in identifying methods to maximize the benefits of Treg adoptive transfer and in generating hypotheses on the key immune dynamics that govern its outcome and that can be tested experimentally. However, as demonstrated in this study and by experimental evidence to date, Treg adoptive transfer alone is insufficient to prevent transplant rejection. These results highlight the need to understand what additional perturbations to the system would better support or enhance the protective function of Tregs. Our mathematical model provides the framework into which treatments like immunosuppression (existing or hypothetical) can be included and used to dissect their very complex effects. Combined with the promising technological advances in both the investigation and manipulation of cells, there is tangible optimism toward the ultimate goal of optimizing therapeutic strategies for transplantation.

## Data Availability

The original contributions presented in the study are included in the article/Supplementary Material, further inquiries can be directed to the corresponding authors.

## Abbreviations

APC, antigen-presenting cell; AT, adoptive transfer; CAR, chimeric antigen receptors; DC, dendritic cell; IL-2, interleukin 2; LN, lymph node; ODE, ordinary differential equation; POD, post-operative day; Tregs, regulatory T cells.
