# Supplementary material for: Modeling the Potential of Treg-Based Therapies for Transplant Rejection: Effect of Dose, Timing, and Accumulation Site
Source: Transpl Int. 2022 Apr 11;35:10297. doi: 10.3389/ti.2022.10297 (PMC9035492; doi:10.3389/ti.2022.10297)
Supplement: Supplementary file 1 [file DataSheet1.PDF]

Supplemental Material

Model Equations.

The model presented in this study is adapted from our original mathematical model of murine heart transplant rejection (1). This compartmental model assumes that all interactions occur in either the graft or the (representative) draining lymph node (Figure S1). The model equations for CD4 T cells (T_H^{LN} and T_H^G), CD8 T cells (T_E^{LN} and T_E^G), inflammatory macrophages (A_{inf}), mature dendritic cells (DCs) in the lymph node (A_{mat}^{LN}), pro- and anti-inflammatory cytokines (C_p and C_a), and graft cells (G) are taken directly from our original model (1). As in (1), naïve CD4 and CD8 T cells (T_{EN}^{LN} and T_{HN}^{LN}) are assumed to remain constant in the model. Transplantation corresponds to the following initial conditions: G(0) = 5,600,000 cells (2, 3), $C_p(0)$ = 50 pg/ml, and A_{imm} = 2000 cells.

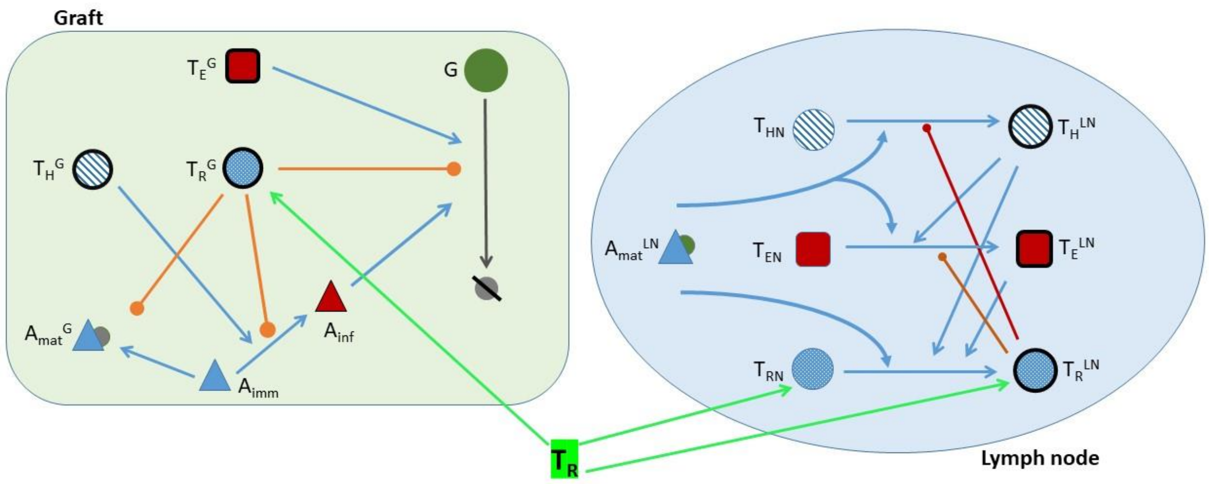

Figure S1. Schematic of transplant-immune interactions defined in the theoretical model with the addition of adoptively transferred regulatory T cells. Compartments for the graft (green) and representative lymph node (blue) are shown. Interactions of the following model populations are depicted: A_{imm}^G, A_{inf}^G, A_{mat}^G, A_{mat}^{LN}, T_{EN}, T_{HN}, T_{RN}, T_E^{LN}, T_H^{LN}, T_R^{LN}, T_E^G, T_H^G, T_R^G, and G. Note, the depictions of C_a^G and C_p^G are not included in the figure to prevent overcrowding the schematic. Blue lines (with arrow tips) indicate activation steps, and red lines (with blunted tips) indicate inhibition steps. Adoptive transfer of Tregs is highlighted in bright green. Adoptive transfer increases the number of activated Tregs in the graft, activated Tregs in the lymph node, and/or naïve Tregs in the lymph node. The schematic is adapted directly from our original model (1).

$$\frac{dT_E^{LN}}{dt} = \frac{a_E T_{EN} A_{mat}^{LN} T_H^{LN}}{(\gamma_1 + A_{mat}^{LN})(\alpha_1 + T_R^{LN})} - \mu_E T_E^{LN} + \frac{r_E T_E^{LN} A_{mat}^{LN}}{\beta_1 + A_{mat}^{LN}} - e_E T_E^{LN} \tag{S1}$$

$$\frac{dT_H^{LN}}{dt} = \frac{a_H T_{HN} A_{mat}^{LN}}{(\gamma_3 + A_{mat}^{LN})(\alpha_3 + T_R^{LN})} - \mu_H T_H^{LN} + \frac{r_H T_H^{LN} A_{mat}^{LN}}{\gamma_4 + A_{mat}^{LN}} - e_H T_H^{LN} \tag{S2}$$

$$\frac{dT_E^G}{dt} = ke_E T_E^{LN} - \mu_E T_E^G + r_{EG}\left(1 - \frac{{C_a^G}^2}{{\eta_3}^2 + {C_a^G}^2}\right)\frac{T_E^G G^G}{\beta_2 + G^G} \tag{S3}$$

$$\frac{dT_H^G}{dt} = ke_H T_H^{LN} - \mu_H T_H^G + r_{HG}\left(1 - \frac{{C_a^G}^2}{{\eta_4}^2 + {C_a^G}^2}\right)\frac{T_H^G A_{mat}^G}{\gamma_5 + A_{mat}^G} \tag{S4}$$

$$\frac{dA_{mat}^{LN}}{dt} = e_A A_{mat}^G - \mu_A A_{mat}^{LN} \tag{S5}$$

$$\frac{dA_{inf}^G}{dt} = a_{p2}\left(1 - \frac{{C_a^G}^2}{{\eta_2}^2 + {C_a^G}^2}\right)\left(\frac{A_{imm}^G T_H^G}{\alpha_5 + T_R^G}\right) - \mu_{Ainf} A_{inf}^G \tag{S6}$$

$$\frac{dC_p^G}{dt} = \frac{\rho_1 A_{mat}^g}{\alpha_9 + T_R^G} + \frac{\rho_2 T_E^G}{\alpha_{10} + T_R^G} + \frac{\rho_3 T_H^G}{\alpha_{11} + T_R^G} + \frac{\rho_4 A_{inf}^G}{\alpha_{12} + T_R^G} - \mu_{C_p} C_p^G \tag{S7}$$

$$\frac{dC_a^G}{dt} = \xi_1 A_{mat}^G + \xi_2 T_R^G + \xi_3 C_p^G G^G + \xi_4 A_{inf}^G - \mu_{C_a} C_a^G \tag{S8}$$

$$\frac{dG^G}{dt} = -\frac{d_{inf} A_{inf}^G G^G}{(\alpha_7 + T_R^G)} - \frac{d_E T_E^G G^G}{(\alpha_8 + T_R^G)} \tag{S9}$$

The equations for immature DCs (A_{imm}^G) and mature DCs (A_{mat}^G) in the graft have been updated to represent experimental observations more accurately. Specifically, the decay rate of immature DCs now is assumed to depend on the remaining graft mass (Eq. S10, second term). Additionally, the activation of immature to mature DCs is assumed to occur in the presence of pro-inflammatory cytokines or CD4 T cells (Eq. S10, third term; Eq. S11, first term).

$$\frac{dA_{imm}^G}{dt} = k_{C_p} C_p^G G^G - \mu_{Aimm}\left(A_{imm}^G - \frac{A_0 G^G}{G_0}\right) - a_{p1}\left(1 - \frac{(C_a^G)^2}{{\eta_1}^2 + (C_a^G)^2}\right)\left(\frac{A_{imm}^G (C_p^G + \zeta T_H^G)}{\alpha_4 + T_R^G}\right)$$
$$- a_{p2}\left(1 - \frac{(C_a^G)^2}{{\eta_2}^2 + (C_a^G)^2}\right)\left(\frac{A_{imm}^G T_H^G}{\alpha_5 + T_R^G}\right) \tag{S10}$$

$$\frac{dA_{mat}^G}{dt} = a_{p1}\left(1 - \frac{(C_a^G)^2}{{\eta_1}^2 + (C_a^G)^2}\right)\left(\frac{A_{imm}^G (C_p^G + \zeta T_H^G)}{\alpha_4 + T_R^G}\right) - \mu_A A_{mat}^G - e_A A_{mat}^G \tag{S11}$$

A dosing function, $D(t)$, for Treg adoptive transfer is defined in Eq. S12 and is added to the equations tracking all Treg populations (Eq. S13-S15). Parameters f_G, f_{LN}, and f_N correspond to the fraction of the Treg dose that enters the graft as activated Tregs, the lymph node as activated Tregs, and the lymph node as naïve Tregs, respectively. In the absence of adoptive transfer, the population of naïve Tregs (T_{RN}^{LN})remains constant at the initial value T_0. Figure S1 provides an updated schematic of the model equations when adoptive transfer of Tregs is introduced into the system (bright green).

$$D(t) = D_0 \sum_{i=1}^{n} d(t) \text{ , where } d(t) = \begin{cases} 0\,, & t < t_i \\ e^{-\beta(t-t_i)}\,, & t \geq t_i \end{cases} \qquad \text{(S12)}$$

$$\frac{dT_R^G}{dt} = ke_R T_R^{LN} - \mu_R T_R^G + \frac{r_{RG} T_R^G (T_E^G + T_H^G)}{\alpha_6 + T_R^G} + f_G D(t) \qquad \text{(S13)}$$

$$\frac{dT_R^{LN}}{dt} = \frac{a_R T_{RN}^{LN} A_{mat}^{LN}}{\gamma_2 + A_{mat}^{LN}} - \mu_R T_R^{LN} + \frac{r_R T_R^{LN} (T_E^{LN} + T_H^{LN})}{\alpha_2 + T_R^{LN}} - e_R T_R^{LN} + f_{LN} D(t) \qquad \text{(S14)}$$

$$\frac{dT_{RN}^{LN}}{dt} = \mu_{RN} (T_0 - T_{RN}^{LN}) + f_N D(t) \qquad \text{(S15)}$$

Table S1. Initial values for model variables. Adapted from Table 2 in our previous work (1).

Variable	Initial Value	Unit	Location
A_{mat}^{LN}	*0*	*cells*	Lymph node
T_E^{LN}	*0*	*cells*	
T_R^{LN}	*0*	*cells*	
T_H^{LN}	*0*	*cells*	
T_{RN}^{LN}	*9500*	*cells*	
A_{mat}^{G}	*200*	*cells*	Graft
A_{imm}^{G}	*2000*	*cells*	
A_{inf}^{G}	*0*	*cells*	
T_E^{G}	*0*	*cells*	
T_R^{G}	*0*	*cells*	
T_H^{G}	*0*	*cells*	
G^G	*5.6e6*	*cells*	
C_p^G	*50*	*pg/mL*	
C_a^G	*0*	*pg/mL*	

Table S2. Names, values, units, and citations for all model parameters. Adapted from Table 3 in our previous work (1). Parameters introduced in this work are in bold. Parameters optimized in this work are classified accordingly.

Parameter Name	Value	Unit	Source
e_A	*5.5*	*1/day*	*(4)*
μ_A	*1.2*	*1/day*	*(5)*
T_{EN}	*55000*	*cells*	*(1)*
a_E	*3*	*1/day*	*(6)*
γ_1	*100*	*cells*	*(6)*
α_1	*2500*	*cells*	*(1)*
μ_E	*0.7*	*1/day*	*(6)*
r_E	*1.51*	*1/day*	*(6)*
β_1	*5000*	*cells*	*(1)*
e_E	*0.001*	*1/day*	*(4)*
$\boldsymbol{T_0}$	***9500***	***cells***	***(1)***
a_R	*1.870e-4*	*1/day*	*optimized*
γ_2	*1000*	*cells*	*(1)*
μ_R	*0.7*	*1/day*	*(4), (7)*
r_R	*0.02*	*1/day*	*(1)*
α_2	*9500*	*cells*	*(1)*
e_R	*0.001*	*1/day*	*(4)*
T_{HN}	*70000*	*cells*	*(1)*
a_H	*4858.45*	*cells/day*	*optimized*
γ_3	*100*	*cells*	*(6)*
α_3	*2500*	*cells*	*(1)*
μ_H	*0.4*	*1/day*	*(4), (6)*
r_H	*1.51*	*1/day*	*(6), (8)*
γ_4	*4000*	*cells*	*(6)*
e_H	*0.001*	*1/day*	*(4)*
a_{p1}	*2432.25*	*cells/day/(pg/ml)*	*optimized*
η_1	*10*	*pg/ml*	*(1)*
α_4	*12000*	*cells*	*(1)*
k_{C_P}	*0.00273*	*1/day/(pg/ml)*	*optimized*
μ_{Aimm}	*106.82*	*1/day*	*optimized*
$\boldsymbol{A_0}$	***2000***	***cells***	***(1)***
a_{p2}	*7.67*	*1/day*	*optimized*
η_2	*10*	*pg/ml*	*(1)*
α_5	*12000*	*cells*	*(1)*
μ_{Ainf}	*1.2*	*1/day*	*(5)*
k	*15*	*-*	*(1)*
r_{EG}	*0.47*	*1/day*	*optimized*

η_3	*10*	*pg/ml*	*(1)*
β_2	*4e6*	*cells*	*(1)*
r_{RG}	*0.002*	*1/day*	*optimized*
α_6	*12000*	*cells*	*(1)*
r_{HG}	*0.755*	*1/day*	*(1)*
γ_5	*4000*	*cells*	*(1)*
η_4	*10*	*pg/ml*	*(1)*
$\boldsymbol{\mu_{RN}}$	***0.002***	***1/day***	*(6)*
d_{inf}	*0.0595*	*1/day*	*optimized*
α_7	*12000*	*cells*	*(1)*
d_E	*0.0043*	*cells/day*	*optimized*
α_8	*12000*	*cells*	*(1)*
ρ_1	*13.99*	*(pg/ml)/day*	*optimized*
α_9	*12000*	*cells*	*(1)*
ρ_2	*0.024*	*(pg/ml)/day*	*(1)*
α_{10}	*12000*	*cells*	*(1)*
ρ_3	*0.24*	*(pg/ml)/day*	*(1)*
α_{11}	*12000*	*cells*	*(1)*
ρ_4	*6.72*	*(pg/ml)/day*	*optimized*
α_{12}	*12000*	*cells*	*(1)*
μ_{C_p}	*0.18*	*1/day*	*optimized*
ξ_1	*2.08e-4*	*(pg/ml)/cells/day*	*(1)*
ξ_2	*6.3e-6*	*(pg/ml)/cells/day*	*(1)*
ξ_3	*4.2e-9*	*1/cells/day*	*(1)*
ξ_4	*2.5e-4*	*(pg/ml)/cells/day*	*(1)*
μ_{C_a}	*0.05*	*1/day*	*(1)*
$\boldsymbol{D_0}$	***[0, 1e7]***	***cells/day***	***varied***
$\boldsymbol{f_G}$	***[0, 1]***	**-**	***varied***
$\boldsymbol{f_{LN}}$	***[0, 1]***	**-**	***varied***
$\boldsymbol{f_N}$	***[0, 1]***	**-**	***varied***
$\boldsymbol{t_i}$	***[0, 20]***	***days***	***varied***
$\boldsymbol{\beta}$	***2***	***1/day***	***assumption***

Model validation.

The updated model yields results that are consistent with our previous experimental observations and retains all expected transplant rejection behavior, while also rectifying unusual behavior seen in the original model. Importantly, the immature DCs no longer decay to zero, as observed *in vivo* (not shown). While the number of antigen-presenting cells (APCs) in the graft has decreased, model predictions for the number of mature DCs remains close to experimental observations (Fig. S2A). In Fig. S2B, the revisions also eliminate the unexpected behavior seen in the original model; the model now predicts that introducing T cells at various days into a system always leads to graft rejection (even when introducing T cells on POD50), which follows experimental expectations (9). All of the model population dynamics predicted by the updated model equations are shown in Fig. S3.

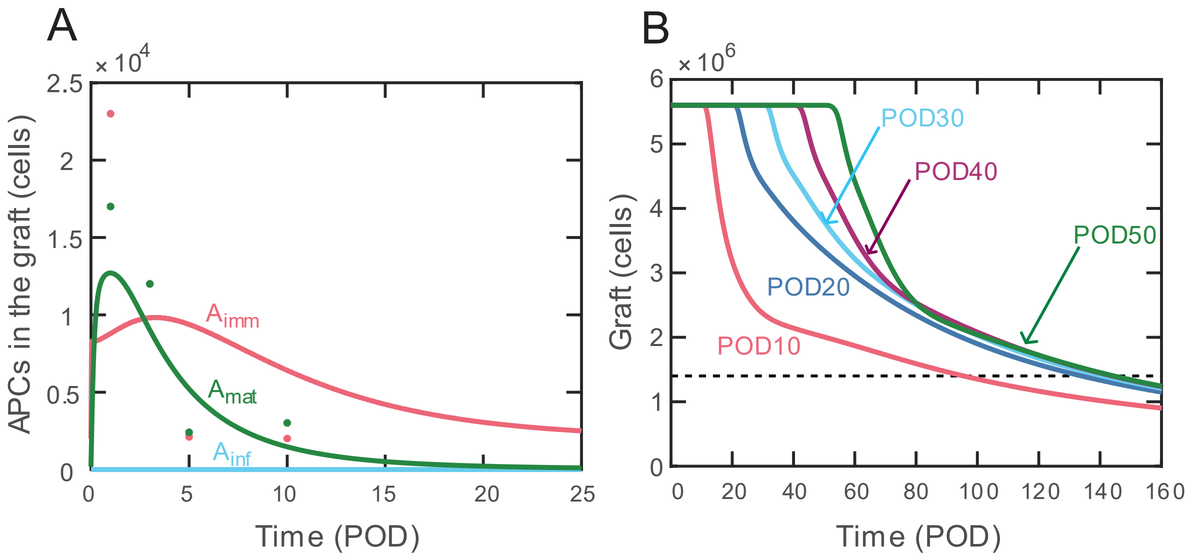

Figure S2. Replication of Fig. 4B and Fig. 7A from our previous work (1) with revised mature and immature DC behavior. **(A)** Model for three APC populations in the graft when no T cells are present: mature DCs (A^G_{mat}, green), immature DCs (A^G_{imm}, magenta), and inflammatory macrophages (A^G_{inf}, cyan). Predictions are compared to datapoints given by (10, 11). **(B)** Graft mass over time when T cells are reintroduced on POD10 (red), POD20 (blue), POD30 (cyan), POD40 (magenta) or POD50 (green).

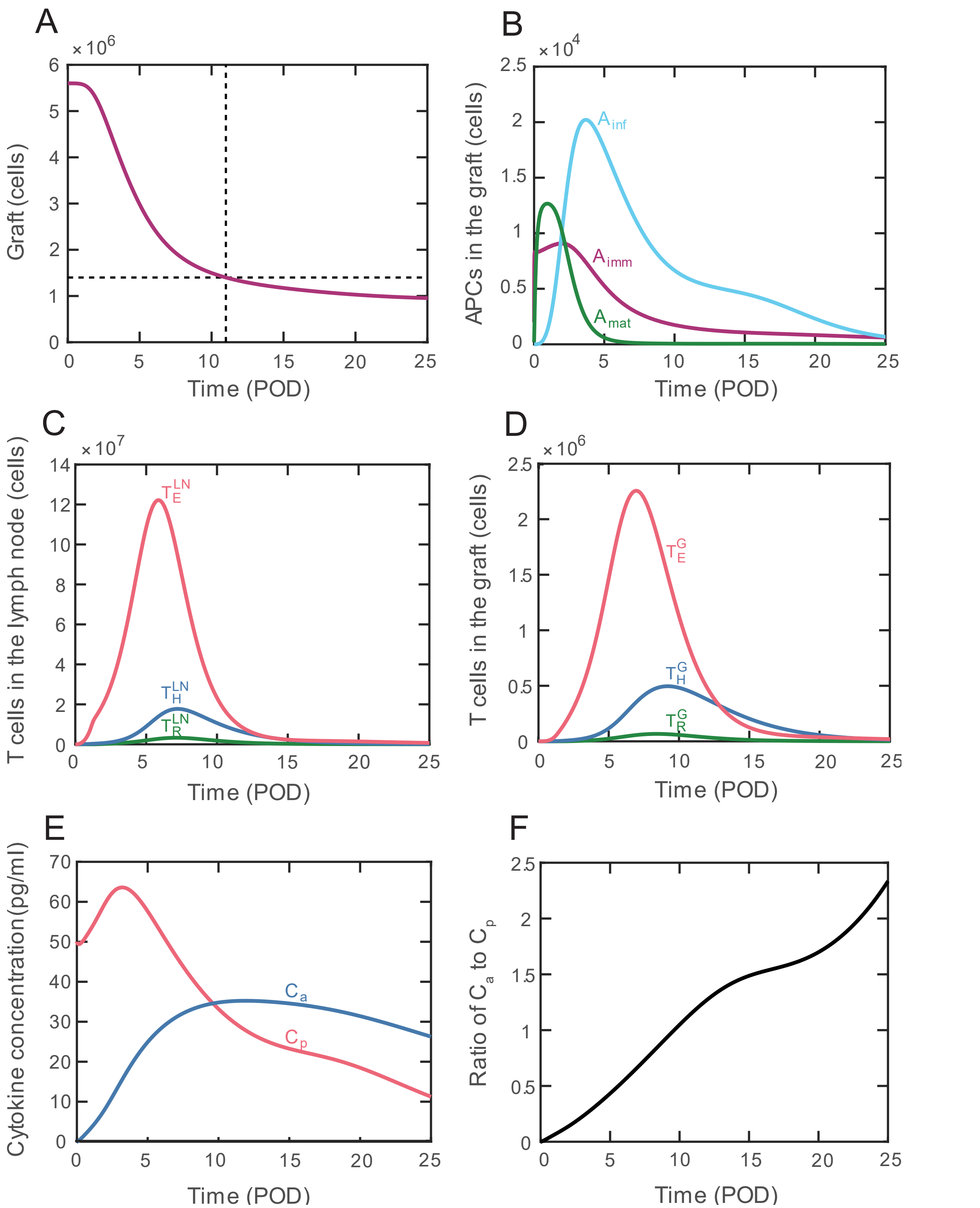

Figure S3. Replication of Fig. 2 from our previous work (1) with revised mature and immature DC behavior. **(A)** Graft rejection is predicted to occur ~11 days post transplantation. **(B)** Model predicted values for three APC populations: immature DCs (A_{imm}^{G}, magenta), mature DCs (A_{mat}^{G}, green), and inflammatory macrophages (A_{inf}^{G}, blue) in the graft. **(C)** Model predicted values for regulatory (T_R^{LN}, green), helper (T_H^{LN}, blue) and effector (T_E^{LN}, red) T cells in the lymph node. **(D)** Model predicted values for regulatory (T_R^{LN}, green), helper (T_H^{LN}, blue) and effector (T_E^{LN}, red) T cells in the graft. **(E)** Model predicted concentrations of anti-inflammatory (C_a, red) and pro-inflammatory (C_p, blue) cytokines in the graft. **(F)** Ratio of $C_a^G : C_p^G$.

Pre-activated vs. naïve Treg adoptive transfer.

The adoptive transfer of pre-activated Tregs is much more effective at protecting the graft than naïve Tregs (Fig. S4). This confirms the benefit of using pre-activated Tregs, which is the same condition deriving from the ex-vivo expansion of Tregs.

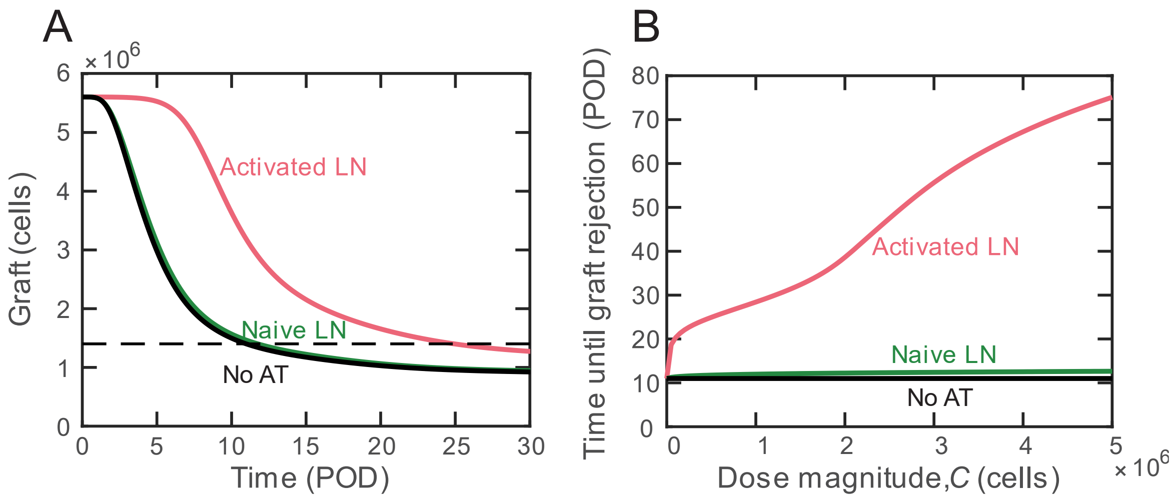

Figure S4. Impact of Treg activation status. **(A)** Number of graft cells shown over time for three different cases: activated Tregs administered to the lymph node (red), naïve Tregs administered to the lymph node (green), and no Tregs administered (black). Tregs are administered on POD0 with a dose magnitude of $C = 5 \times 10^5$ cells. The horizontal dashed line indicates a 75% reduction in initial graft size. The intersections of the curves with this dashed line give the model predicted values of graft rejection time. **(B)** Model predicted rejection time as the dose magnitude (C) is varied for a single Treg injection on POD0. Tregs are delivered to the lymph node as activated (red) or naïve (green) cells. The black line marks POD11, which is when rejection occurs without adoptive transfer.

Impact of accumulation site on host immune dynamics.

According to the model, activated Tregs that accumulate in the graft are more effective at prolonging graft lifetime than activated Tregs that accumulate in the lymph node for almost all dosing rates (Fig. 2). Tregs in the graft inhibit DCs from maturing (Fig. S5B) and subsequently translocating to the lymph node to activate T cells. This proves much more effective at limiting activation of cytotoxic CD8 T cells and inflammatory macrophages than Tregs that accumulate in the lymph node (Fig. S5C-D), and therefore destruction of the transplant occurs more slowly in this scenario (Fig. S5E-F).

Impact of dose timing on host immune dynamics.

When modeling a single dose of 5e5 Tregs, the non-monotonic relationship between dose timing and graft survival indicates that it is best to inject Tregs on POD1.5. The reason for this is similar to that given for varying dose frequency in Section 3.4. Adoptive transfer on POD1.5 times cell delivery to coincide with the surge of cytotoxic CD8 T cells and inflammatory macrophages, while also ensuring there are Tregs in the graft soon after transplantation to minimize DC maturation and T cell activation (Fig. S6).

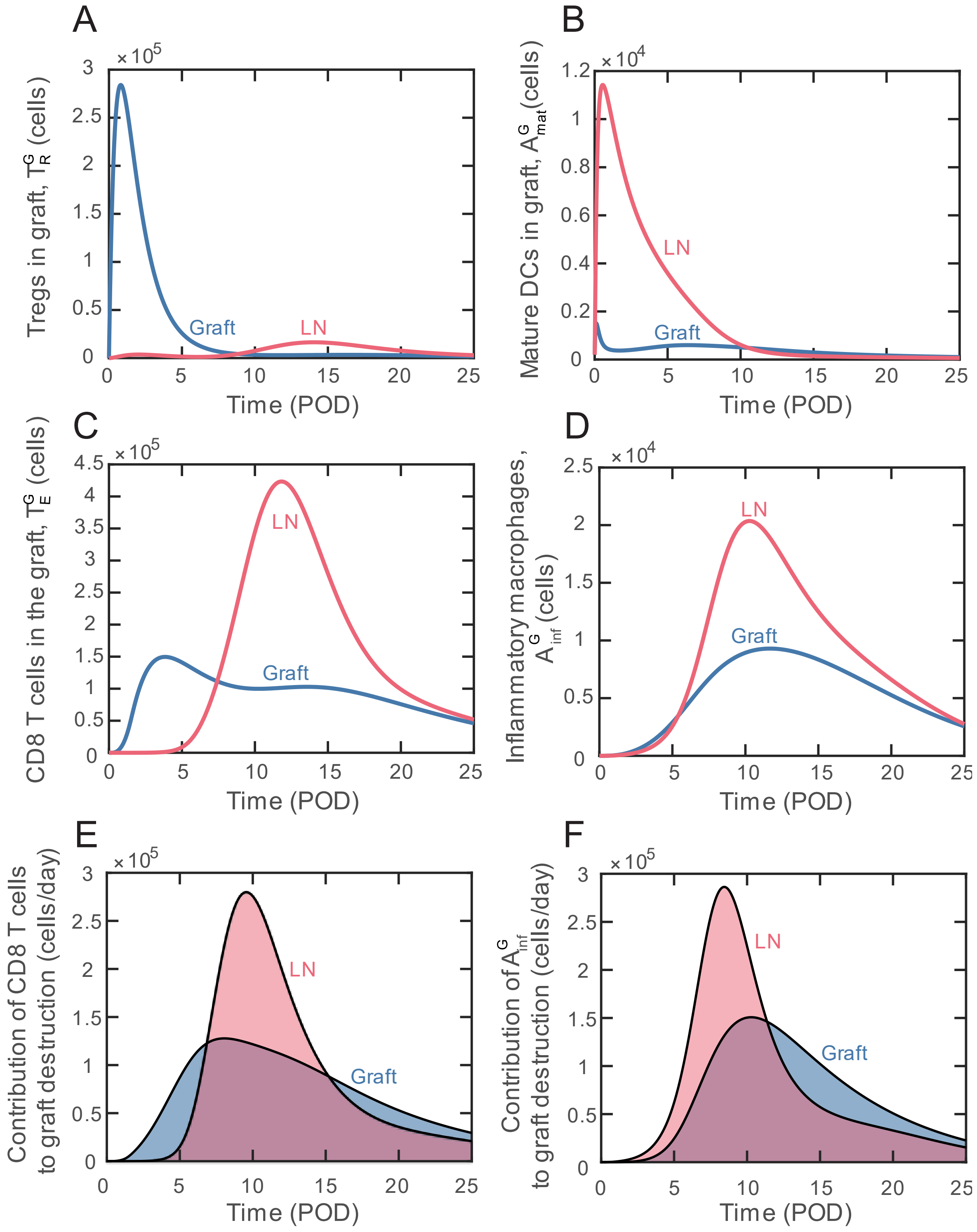

Figure S5. Model predicted values for host immune dynamics with $C = 5 \times 10^5$ activated Tregs delivered to the graft (blue) or lymph node (red). **(A)** Number of Tregs in the graft, T_R^G. **(B)** Number of mature DCs in the graft, A_{mat}^G. **(C)** Number of cytotoxic CD8 T cells in the graft, T_E^G. **(D)** Number of inflammatory macrophages in the graft, A_{inf}^G. **(E)** Rate of destruction caused by cytotoxic CD8 T cells. **(F)** Rate of destruction caused by inflammatory macrophages.

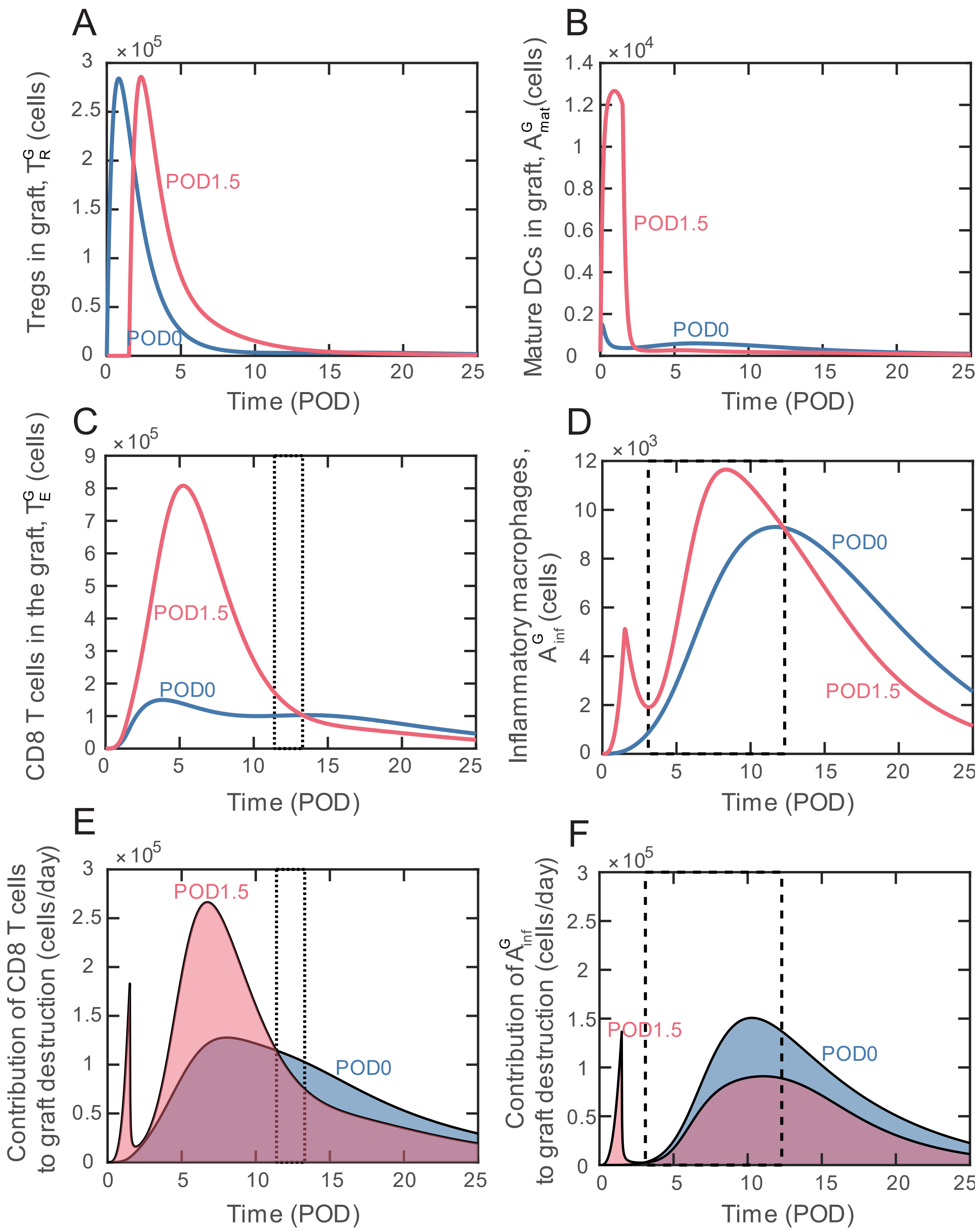

Figure S6. Model predicted values for host immune dynamics with a single dose of $C = 5 \times 10^5$ Tregs delivered on POD0 (blue) or POD1.5 (red). Dashed boxes highlight timeframes when destructive cell quantity is higher but rate of destruction is lower for POD1.5 treatment. **(A)** Number of Tregs in the graft, T_R^G. **(B)** Number of mature DCs in the graft, A_{mat}^G. **(C)** Number of cytotoxic CD8 T cells in the graft, T_E^G. **(D)** Number of inflammatory macrophages in the graft, A_{inf}^G. **(E)** Rate of destruction caused by cytotoxic CD8 T cells. **(F)** Rate of destruction caused by inflammatory macrophages.

References

1. Arciero JC, Maturo A, Arun A, Oh BC, Brandacher G, Raimondi G. Combining Theoretical and Experimental Techniques to Study Murine Heart Transplant Rejection. Front Immunol. 2016;7:448.
2. Doevendans PA, Daemen MJ, de Muinck ED, Smits JF. Cardiovascular phenotyping in mice. Cardiovascular research. 1998;39(1):34-49.
3. Tirziu D, Giordano FJ, Simons M. Cell communications in the heart. Circulation. 2010;122(9):928-37.
4. Kim PS, Lee PP, Levy D. Modeling regulation mechanisms in the immune system. Journal of theoretical biology. 2007;246(1):33-69.
5. Day J, Rubin J, Vodovotz Y, Chow CC, Reynolds A, Clermont G. A reduced mathematical model of the acute inflammatory response II. Capturing scenarios of repeated endotoxin administration. J Theor Biol. 2006;242(1):237-56.
6. Lee HY, Topham DJ, Park SY, Hollenbaugh J, Treanor J, Mosmann TR, et al. Simulation and prediction of the adaptive immune response to influenza A virus infection. Journal of virology. 2009;83(14):7151-65.
7. Kronik N, Kogan Y, Elishmereni M, Halevi-Tobias K, Vuk-Pavlovic S, Agur Z. Predicting outcomes of prostate cancer immunotherapy by personalized mathematical models. Plos One. 2010;5(12):e15482.
8. De Boer RJ, Homann D, Perelson AS. Different Dynamics of CD4⁺ and CD8⁺ T Cell Responses During and After Acute Lymphocytic Choriomeningitis Virus Infection. The Journal of Immunology. 2003;171(8):3928-35.
9. Bingaman AW, Ha J, Waitze SY, Durham MM, Cho HR, Tucker-Burden C, et al. Vigorous allograft rejection in the absence of danger. J Immunol. 2000;164(6):3065-71.
10. Oberbarnscheidt MH, Zeng Q, Li Q, Dai H, Williams AL, Shlomchik WD, et al. Non-self recognition by monocytes initiates allograft rejection. J Clin Invest. 2014;124(8):3579-89.
11. Su CA, Iida S, Abe T, Fairchild RL. Endogenous memory CD8 T cells directly mediate cardiac allograft rejection. Am J Transplant. 2014;14(3):568-79.
